# Assessment of Hereditary Retinal Degeneration in the English Springer Spaniel Dog and Disease Relationship to an *RPGRIP1* Mutation

**DOI:** 10.1155/2012/685901

**Published:** 2012-02-28

**Authors:** Kristina Narfström, Manbok Jeong, Jennifer Hyman, Richard W. Madsen, Tomas F. Bergström

**Affiliations:** ^1^Department of Veterinary Medicine and Surgery, College of Veterinary Medicine, University of Missouri, Columbia, MO 65211, USA; ^2^Department of Ophthalmology, Mason Eye Institute, University of Missouri, Columbia, MO 65211, USA; ^3^Department of Veterinary Medicine and Surgery, College of Veterinary Medicine and BK21 Program for Veterinary Science, Seoul National University, Seoul 151-742, Republic of Korea; ^4^Eye Care For Animals, VA, 20176-3367, USA; ^5^Biostatistics, School of Medicine, University of Missouri, Columbia, MO 65211, USA; ^6^Department of Animal Breeding and Genetics, Swedish University of Agricultural Sciences, 750 07 Uppsala, Sweden

## Abstract

Intensive breeding and selection on desired traits have produced high rates of inherited diseases in dogs. Hereditary retinal degeneration, often called progressive retinal atrophy (PRA), is prevalent in dogs with disease entities comparable to human retinitis pigmentosa (RP) and Leber's congenital amaurosis (LCA). Recent molecular studies in the English Springer Spaniel (ESS) dog have shown that PRA cases are often homozygous for a mutation in the RPGRIP1 gene, the defect also causing human RP, LCA, and cone rod dystrophies. The present study characterizes the disease in a group of affected ESS in USA, using clinical, functional, and morphological studies. An objective evaluation of retinal function using electroretinography (ERG) is further performed in a masked fashion in a group of American ESS dogs, with the examiner masked to the genetic status of the dogs. Only 4 of 6 homozygous animals showed clinical signs of disease, emphasizing the need and importance for more precise studies on the clinical expression of molecular defects before utilizing animal models for translational research, such as when using stem cells for therapeutic intervention.

## 1. Introduction

The domestic dog has a unique population history with bottlenecks that has shaped the diversity and structure of the canine genome. The first bottleneck can be traced back about 7,000–50,000 generations [1] and reflects the early domestication of dogs from wild populations of wolves 15,000–100,000 years ago [2–4]. When dog breeds were established in the 1800s (about 50–100 generations ago) more genetic variation was lost, and this second population bottleneck resulted in relatively large genetic differences between breeds and little genetic variation within breeds. The two bottlenecks left distinctive signatures in the canine genome with long-range linkage disequilibrium (LD) and long haplotype blocks of 500 kb–1 Mb within breeds and short-range LD across breeds [1, 5]. Intensive breeding and selection on desired traits have also produced high rates of inherited diseases with genetic causes that are breed-specific or nearly so. The domestic dog has therefore emerged as an important animal model for comparative genome analysis and for characterization of inherited disease. Other uses for dog models are in translational research such as gene therapy or stem cell transplantation for treatment strategies in conjunction with hereditary retinal blinding diseases.

Hereditary retinal degeneration, often called progressive retinal atrophy (PRA) in dogs and also in cats, is a group of diseases of the photoreceptors that exists in various forms. Rods and cones are usually primarily affected, with time leading to bilateral blindness. More than 100 dog breeds are on the list for those that may be affected, in which at least 15 mutations are prevalent in 34 specific dog breeds [6]. For cats, PRA is observed less frequently, although recently, a mutation in the CEP290 gene was shown to be causative of hereditary retinal degeneration in a large number of cat breeds, primarily Abyssinian and Siamese cats [7].

Through advancement in the understanding of hereditary disease processes affecting the outermost portion of the retina, PRA has been further characterized biochemically, electrophysiologically, morphologically, and genetically. Using molecular methods, including the elucidation of causative mutant genes for several hereditary retinal disorders, much knowledge has been gained especially in regards to disease mechanisms [8]. The availability of the canine genome sequence [1] (http://www.genome.gov/12511476) has simplified the task of identifying genes responsible for diseases and traits in dogs. Similarly, a full genome sequence (10X) of the cat has recently been completed (Wes Warren, Washington University, personal communication, 2010). A number of the PRAs have been designated gene symbols reflecting either the specific cells involved in the hereditary retinal dystrophy or the protein involved in the retinal degenerative condition.

Although PRA is usually manifested with a breed specific phenotype, the same allelic mutation may be shared by several different breeds, such as for the *prcd* mutation [9]. In other instances, different but allelic mutations causative of PRA have been documented in related breeds [10]. Importantly, breeds may also express more than one form of PRA. For example, in golden retrievers at least three different genes and mutations are responsible for PRA. Two of the genes are known, prcd and slc4a3, but at least one more gene remains to be characterized [11]. The presence of more than one causative mutation for PRA in some breeds is complicating the understanding and interpretation of phenotype-genotype relationships and thus the results of DNA testing procedures [12]. Due to these factors, phenotypic heterogeneity is often found when studying various forms of retinal degenerative diseases of dogs. This has also been observed in similar retinal disease processes of humans, such as in retinitis pigmentosa (RP) or in Leber's congenital amaurosis (LCA). In these diseases, significant phenotypic heterogeneity is found including age of onset, clinical findings, and progression of disease [8, 13]. This is especially true for RP in which at least 140 mutations have been described only in the rhodopsin gene [14, http://omim.org/entry/268000].

Classical PRA has been described as a generalized disease of the fundus, affecting the rod photoreceptors primarily and with later involvement of cones. Clinically night blindness is an early sign but with progression of disease, affection of both night and day vision occurs. Ophthalmoscopically early changes have been noted as a generalized diffuse change in coloration and reflectivity of the tapetal fundus with vascular attenuation in later stages, the changes being most severe in the peripheral parts but with later involvement of the entire fundus [15].

PRA in the English Springer Spaniel dog was originally described in USA [16]. There were some unusual findings in the disease in that there was an increased granularity of the fundus or a slightly patchy discoloration as the earliest clinical sign observed in the very far periphery of the tapetal fundus. The disease had a variable time of onset and progression, leading to blindness in most of the affected dogs.

Mutations in the RPGRIP1 gene have been identified as causative to human RP, LCA, and cone-rod dystrophies [17–19]. The RPGRIP1 protein was shown to be involved in transport mechanisms that occur through the photoreceptor connecting cilium by interaction with another protein, RPGR [20]. Defects in the latter gene are responsible for the X-linked retinopathy of humans and also in X-linked PRA in Samoyed and Siberian husky dogs [21]. Further, a mutation in NPHP4, another gene known to interact with RPGRIP1, was shown to be causative of cone/rod dystrophy of the wire-haired Dachshund [22, 23]. Another such gene working in concert with RPGRIP1 is CEP290, and when mutated it was found to cause hereditary retinal degeneration in cats [24]. The protein is also an important component of the transport mechanism, whereby specialized proteins critical for phototransduction are transferred from their site of synthesis in the inner segment through the connecting cilium to the outer segment [25]. A number of normal genes are thus necessary for normal function and structure of the entire photoreceptor cell and, especially, for outer segment disc morphogenesis [26].

Mellersh and collaborators recently mapped the cone rod locus (cord1) to a 14.5 Mb region on dog chromosome 15 (16.54–30.68 Mb: coordinates as in CanFam2.0), which contained the RPGRIP1 gene. A 44 bp insertion in exon 2 was further identified in the RPGRIP1 gene, truncating the protein. This defect segregated completely with the cord1 phenotype of cone rod dystrophy in the Animal Health Trust (United Kingdom) research colony of miniature dachshunds. It was concluded that the mutation was responsible for cord1 disease, due to a mutation in the RPGRIP1 gene. It was further emphasized that dogs with this disease are valuable animal models for exploring disease mechanisms and potential therapies for the human counterpart, LCA [27].

The *RPGRIP1* mutation in cone rod dystrophy (cord1) was further evaluated as a candidate gene for PRA in ESS dogs using DNA collected at the University of Missouri (Columbia, USA), from a large number of dogs, unaffected and affected by bilateral, generalized retinal degeneration. The mutation was observed in exon 2 of RPGRIP1 in all of the affected dogs (Gary Johnson and Liz Hansen, personal communications, 2007).

This paper describes the results of a research project that was thereafter initiated, in order to characterize the clinical signs of retinal degeneration in the ESS breed and to evaluate the genotype-phenotype correlation in USA in family-owned ESS, in regards to the mutation in the RPGRIP1 gene. Recently, the study was further expanded to also include blood samples from a group of Swedish ESS dogs, with and without clinical signs of PRA and the correlation in regards to the *RPGRIP1* mutation.

## 2. Materials and Methods

### 2.1. Animals and Clinical Examinations

ESS dogs from USA were included in the characterization of disease. They were privately owned dogs for which owners or breeders had requested eye examinations, according to eye scheme routines, since their dogs or their close relatives were used for breeding. Twelve cases with clinical signs of retinal degeneration in 1.5- to 12-year-old ESS dogs were discovered during the two-year study period. All of the affected dogs were homozygous for the *RPGRIP1* mutation.

Informed consent was obtained from the owners of participating dogs. The clinical study included evaluation of retinal and vision based responses and reflexes: menace, dazzle, and examination of the pupillary light reflexes as well as visual testing by behavior and visual reactions to falling cotton balls [28]. Pupils were dilated with short-acting mydriatics 20 minutes before examination of the internal structures, using 1-2 drops of 1% tropicamide in each eye (Mydriacyl, Bausch and Lomb Inc., Tampa, FL). Standard ophthalmic examination of the interior of the eye was then performed using indirect ophthalmoscopy (Welch-Allyn Distributors, Medical Device Depot, Inc., MD, USA) and slit lamp biomicroscopy (SL14, KOWA Co. Ltd., Tokyo, Japan). Fundus appearance was documented with a digital fundus camera (Nidek NM-100, Nidek Co. Ltd., Freemont, CA).

### 2.2. DNA Samples

DNA samples from ESS dogs previously collected by Dr. Gary Johnson's laboratory, University of Missouri, Columbia, USA, were utilized. All samples were collected as surplus from blood specimens submitted for routine *cord1* test or for clinical biochemistry under the condition of anonymity of the individuals and their owners. DNA was extracted from blood samples and genotyping for the *cord1* disease causing allele of the mutated gene RPGRIP1 gene was performed as described by Mellersh et al., 2006 [27].

In addition, to investigate whether the cord1 genotype was prevalent among ESS dogs in Sweden, and especially in ESS dogs diagnosed with PRA, blood samples from a total of 14 normal and affected dogs were tested for the 44 bp insertion in exon 2 of the RPGRIP1 gene (Table 1). The blood from the Swedish dogs was collected into EDTA tubes and genomic DNA was extracted manually from peripheral blood leukocytes using QIAamp DNA Blood Midi Kit (Qiagen, Hilden, Germany) or automatically on a QIA symphony SP/AS instrument (Qiagen, Hilden, Germany).

Primers for genotyping the 44 bp insertion in exon2 of RPGRIP1 gene were designed using the software Primer3 [29]. PCR amplification was performed using the primers Cfa_Cord1-F (5′-6FAM-CCCTTTCCTGGGACTTTAGG-3′) and Cfa_Cord1-R (5′-CCCTCTGCCTATGTCTCTGC-3′). 10–20 ng of genomic DNA was used in a 10 uL PCR-reaction with 0.5 mM of each primer, 50 mM KCl, 10 mM Tris-HCl pH 8.3, 2.5 mM MgCl_2_, 0.2 mM of each dNTP and 0.5 units of Taq polymerase (AmpliTaq Gold; Applied Biosystems, Foster City, CA, USA; DNA polymerase). A total of 35 PCR cycles was performed, each with denaturation at 94°C for 1 minute, annealing at 60°C for 40 s and a primer extension at 72°C for 40 s. The fragment length polymorphism was then determined using an ABI 3100 DNA Analyzer and GeneMapper Software (Applied Biosystems, Inc., (ABI), Foster City, CA).

### 2.3. Masked Electroretinography Study

Fourteen American dogs were included in a masked electroretinography (ERG) study for objective evaluation of retinal function (for ERG see details below). Blood from these dogs, age between 7 y 9 m and 13 y 10 m, had previously been genotyped by Dr. Johnson's laboratory, as described above. Dogs were chosen for retinal functional evaluation in accordance with the owner's consent and availability. The genetic status of each dog in regards to the *RPGRIP1* mutation was unknown to the investigator (K. Narfström) at the time of the ERG recordings: 6 dogs were homozygous (affected); *RPGRIP−/−,* 7 were heterozygous (normal); *RPGRIP−/+*, and 1 was homozygous (normal); *RPGRIP1+/+ *(Table 2). Before the ERG was performed in each dog, they underwent a routine ophthalmic examination as previously described. 

### 2.4. Electroretinography

**Figure 1:**
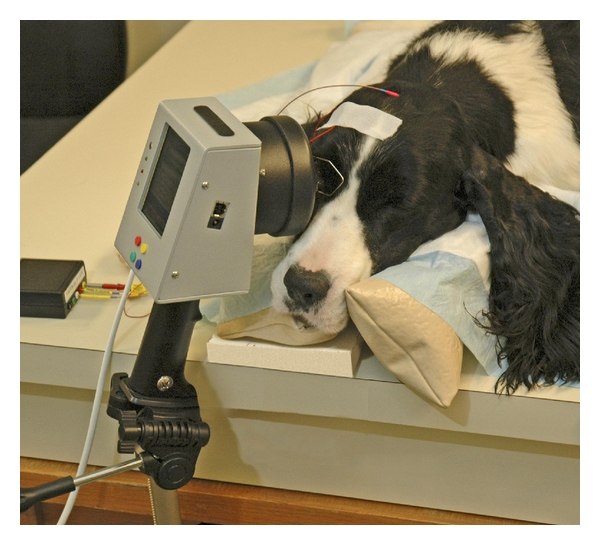
An English Springer Spaniel (ESS) dog deeply sedated and prepared for functional evaluation of the retina. The handheld multispecies electroretinograph (HMsERG) unit is used together with a preprogrammed protocol for evaluation of rod and cone function.

Unilateral electroretinographic (ERG) evaluations were performed using a portable ERG unit (HMsERG model 1000, RetVet Corp., Columbia, MO), with a mini-Ganzfeld dome positioned approximately 1 cm from the right tested eye (Figure 1). For practical reasons it was deemed sufficient to perform the evaluation in only one eye since both eyes are usually affected in hereditary retinopathies and the eyes are usually at the same stage of the retinal degenerative process [15].

Dogs were deeply sedated by using medetomidine IV (Domitor, Novartis, Pfizer Animal Health, Exton, PA), up to 150 micrograms/kg, equivalent to 0.15 mL/kg, and prepared for the ERG session in ordinary room light. Heart and respiratory rates were closely monitored before and throughout the procedure and the dogs were temperature controlled. The dog's head was positioned on a cushion for stabilization. Maximal pupillary dilation was provided for by the use of short-acting mydriatics (see above) and the eye was further topically anesthetized using 0.5% proparacaine hydrochloride (Alcaine, Alcon, Fort Worth, TX). A lid speculum was inserted to ensure that the nictitating membrane as well as the upper and lower eyelids did not interfere with light exposure to the maximally dilated pupils. Platinum subdermal needle electrodes (model E2, Grass Instrument Division, Astro-Med, Inc., West Warwick, RI) were used for the ground electrode, positioned on the occipital crest, and for the reference electrode, positioned 3 cm from the lateral canthus, close to the base of the right ear. An active contact lens electrode (ERG-Jet, Universo Plastique, LKC Technologies Inc., Gaithersburg, Md) was placed on the cornea after instillation of one drop of 2% methylcellulose (Methocel, Ciba Vision, Munich, Germany). The electrodes were connected to a preamplifier and the signals were amplified with a bandpass filter between 0.3 and 300 Hz.

Each ERG session consisted of scotopic and photopic ERGs in accordance with the “Dog Diagnostic Protocol,” recommended by the European College of Veterinary Ophthalmologists, primarily for evaluation and separation of rod and cone function [30]. This protocol is preprogrammed on the ERG unit and is executed automatically upon initiation of the ERG session by the examiner. During 20 minutes of dark adaptation, scotopic-low intensity rod responses were elicited every 4 minutes at a stimulus intensity of 0.01 cd·s/m^2^; responses were averaged after 10 flashes given at 2 seconds interval and rod responses were recorded at each time point (test #1–5). The light stimulus intensity was then increased to 3 cd·s/m^2^ for scotopic standard intensity stimulation and responses averaged and recorded after 4 flashes at 10-second intervals (test #6). Thereafter scotopic high-intensity responses were elicited using 10 cd·s/m^2^; responses were averaged to 4 flashes administered at 20-second intervals (test #7). The latter two recordings depict a mixture of responses from both rods and cones. After 10 minutes of light adaptation with a background luminance of 30 cd/m^2^, photopic single flash responses were recorded, using 3 cd·s/m^2^ of flash stimulus, averaging 32 flashes at an interval of 0.5 seconds (test #8), followed by evaluation of 30 Hz photopic flicker at the same light intensity stimulation (test #9). The latter two recordings were performed in order to evaluate cone and cone pathways, respectively. Data were collected automatically on the compact flash card of the ERG unit, transferred to a computer, printed, and stored for further analysis. ERGs were evaluated and the amplitudes and implicit times for the a- and b-waves were measured as previously described [30].

After termination of the ERG session an injection of atipamazole hydrochloride (Antisedan, Pfizer Inc., St Louis, MO) was administered intramuscularly to reverse the deep sedation (at a dosage 5-times higher than that given of the medetomidine, i.e., similar volumes were injected).

### 2.5. Morphology

Upon the owner's request due to unrelated medical problems, three American ESS dogs were euthanized and the eye tissue made available for the present study. Advanced PRA had been diagnosed in two of the dogs (9 and 6 years old, resp.), while the third dog, 3 years old, had a normal fundus appearance. Euthanasia was performed by intravenous infusion of Beuthanasia-D-Special (Schering Plough Animal Health, Omaha, NE.). The eyes of each dog were enucleated immediately after death and the posterior segment of each eye placed in fixative solution for examination using light and electron microscopy (LM and EM). The fixative included 2.0% glutaraldehyde, 1.12% paraformaldehyde, 0.13 M sodium cacodylate, 0.13 mM CaCl_2_, pH 7.40. Eyecups were incubated with gentle agitation for at least 2 hours at room temperature. The eyecups were then gross sectioned to obtain 2 × 3 mm pieces from the following regions: the central part of the fundus, temporal to the optic nerve head (the area centralis-like region), superior midperiphery and periphery, and inferior midperiphery and periphery. Samples from these regions were postfixed in 1% osmium tetroxide and embedded in epoxy resin. They were washed with 0.17 M sodium cacodylate, pH 7.4, followed by secondary fixation in 1% osmium tetroxide. Subsequently, the samples were dehydrated via sequential incubation in increasing concentrations of acetone and embedded in epoxy resin. Sections of the embedded samples were cut for both LM and EM examinations. For LM, 1-micron-thick sections were mounted on glass slides and stained with toluidine blue. For EM, sections were mounted on copper grids and were stained with uranyl acetate and lead citrate. LM was performed using a Zeiss Axiophot microscope and EM was performed using a JEOL 1200 EX transmission electron microscope.

### 2.6. Statistical Evaluations

Descriptive statistics were performed in relation to the masked ERG study using the SAS v9 (SAS Institute Inc. Cary, NC, USA) of the dogs classified as normal (including homozygous normal and heterozygous dogs) and affected dogs by DNA analysis for the *RPGRIP1* mutation. Due to the small sample size in the affected group and the fact that the data did not show any extreme outliers, two-sample *t*-tests with the Satterthwaite approximation for degrees of freedom allowing for unequal variances between the groups were utilized. Due to the marked differences between the groups, results were considered significant at the 0.01 significance level.

## 3. Results

### 3.1. Clinical Characterization of Retinal Degeneration due to the *RPGRIP1* Mutation

A 10-year-old dog was examined due to severe visual problems reported by the owner, first noted at the age of 8 years. Advanced signs of retinal degeneration were observed by ophthalmoscopy in this dog. None of the other 11 dogs examined with clinical signs of retinal disease (ophthalmoscopic changes or reduced ERG responses) had shown visual problems until the age of 6–9 years, according to the owners.

**Figure 2:**
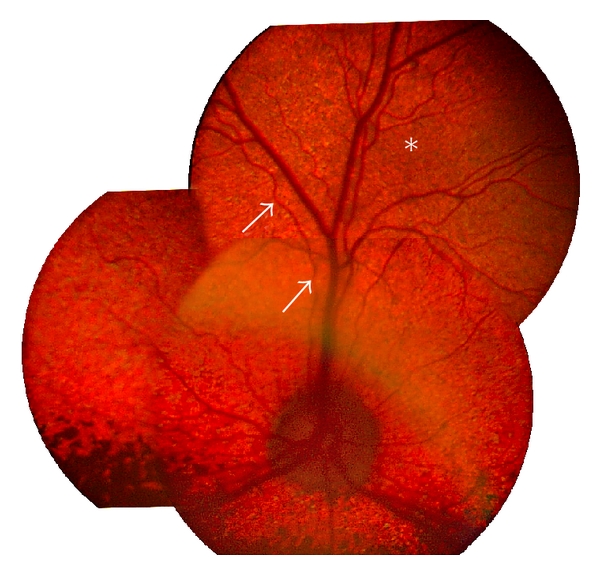
A composite of fundus pictures from a two-year-old ESS dog affected with cone rod dystrophy and homozygous for the *RPGRIP1* mutation. The central fundus is mainly normal appearing while an increase in granularity is observed in the midperipheral and peripheral fundus (star). There is also slight vascular attenuation with variable diameter of retinal vasculature (arrows).

The earliest ophthalmoscopic signs of disease were observed in a 1.5- and a 2-year-old ESS dog, respectively. Both showed increased granularity in the far peripheral tapetal fundus, with minute hyporeflective brown to gray spots in the far periphery of the tapetal fundus (Figure 2). With increasing age (in 3–8-year-old dogs) these abnormalities became more generalized with diffuse mottling of the tapetal fundus and changes in fundus coloration. There was also generalized changes in tapetal reflectivity (hyporeflectivity and with movement of the lens used for indirect ophthalmoscopy, some of these areas became hyperreflective). At this later stage there was also severe attenuation of retinal vasculature. At 9-10 years of age, a generalized, end-stage type of retinal degeneration was observed in most affected dogs with a marked hyperreflective tapetal area, severe attenuation of retinal vasculature with few vessels still visible, mainly in the central parts of the fundus. At this stage there was also decoloration interspersed with hyperpigmentation of the nontapetal fundus. One 9-year-old dog had a mainly normal fundus appearance although ERG examination showed reduced responses for both the cone and the rod system. Bilateral, secondary cataracts (complete and immature types) were observed at age 12 years in one of the clinically affected dogs described in the present study.

### 3.2. Genotype-Phenotype Evaluation in Swedish ESS

Blood from 14 dogs was included in the study in which ten of the dogs had been diagnosed with PRA. Two cases of PRA were found to be homozygous for the disease causing RPGRIP allele (−/−), four were genotyped as heterozygous (+/−) and four of the cases had the homozygous genotype for the normal allele (+/+) (Table 1).

### 3.3. Masked ERG Study

A marked reduction of ERG responses was observed in 4 of 6 of the dogs that had been diagnosed as homozygous for the *RPGRIP1* mutation through blood testing (Table 2). Three of the 4 dogs evaluated as affected by ERGs had not shown any apparent deficiency in vision as evaluated by the owner or by the examiner. Two of these dogs had normal fundus appearances, while the other two dogs had early and moderately advanced retinal degenerative changes, respectively. The two dogs, homozygous for the *RPGRIP *mutation that had normal ERGs, were also ophthalmoscopically normal.

**Figure 3:**
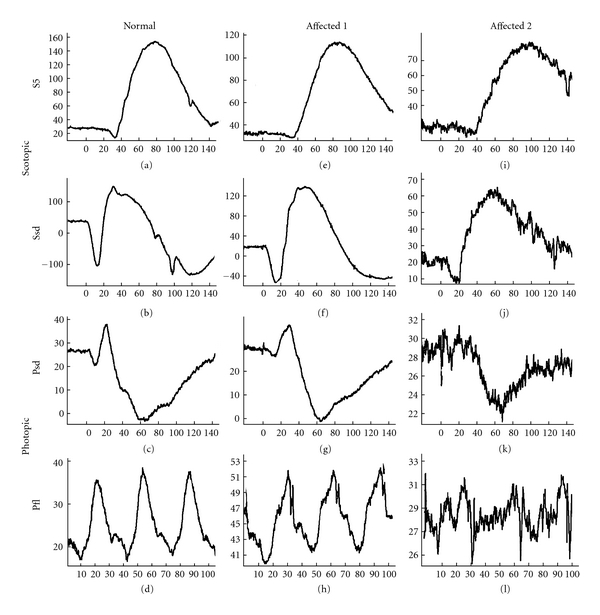
Scotopic and photopic ERGs from a normal 8-year-old ESS dog and two RPGRIP1−/− affected ESS dogs at the age of 9 (affected 1) and 12 years (affected 2), respectively. The latter affected dog was normal at ophthalmoscopic examination when it was 9 years old. At the age of 12 years it was only slightly visually compromised, and in a moderately advanced stage of retinal degeneration as seen by ophthalmoscopy. S5: scotopic ERG response using 10 mcd·s/m^2^ of light stimuli after 20 minutes of dark adaptation, Ssd: scotopic ERG response using 3 cd·s/m^2^ of light stimuli in the dark, Psd: photopic ERG response using 3 cd·s/m^2^ in the light after 10 minutes of light adaptation (with 30 cd/m^2^ of background light), Pfl: photopic ERG flicker response after 30 Hz flickering light stimuli in the light adapted state.

Results of ERG recordings in a normal 8-year-old ESS and in two affected dogs, 9 and 12 year olds, respectively, are shown in Figure 3. Evaluation of a-wave amplitudes showed significant differences between the affected group and the heterozygous and homozygous normal group, at the 0.01 significance level, when scotopic and photopic standard intensity responses were compared (*P* < 0.0004 and *P* < 0.0007, resp.). For the b-wave these differences were also significant at the same level for 4 of the 5 scotopic low-intensity responses evaluated (#2–5) (*P* < 0.0084, 0.0003, 0.0005, and 0.0020, resp.) and for the photopic flicker response (*P* < 0.0032). For a- and b-wave implicit times there were no significant differences for the former while for the latter a significant longer implicit time was found for the affected group of dogs in comparison to the normal group for scotopic standard intensity stimulation (*P* < 0.0066). For ERG amplitude and implicit time details see Table 3.

Two dogs in the group, found to be homozygous for the *RPGRIP1* mutation by blood testing, had a- and b-wave amplitude and implicit time values that were in complete accordance with the normal group of dogs.

In order to evaluate if the cone system was more affected than the rod system by the disease, the percentage reduction for each was calculated. This was performed through comparison of the median response from affected and normal dogs, respectively, using photopic standard intensity (test #8) and photopic flicker responses (test #9) for the cone system and scotopic low-intensity stimulation (test #5) for the rod system. It was found that the cone system was more affected than the rod system: 46% and 58% reduction, respectively, for the cones and 44% reduction for the rods in the affected group when compared with the normal ESS group of dogs.

### 3.4. Morphology

Light and electron microscopies were performed using retinal sections from 3 dogs. The two older dogs had ophthalmoscopic signs of severe bilateral generalized retinal degeneration while the younger, a 3-year-old ESS dog, exhibited a granular but otherwise normal fundus appearance. DNA from the youngest dog only was obtained, and showed homozygocity for the *RPGRIP1* mutation.

**Figure 4:**
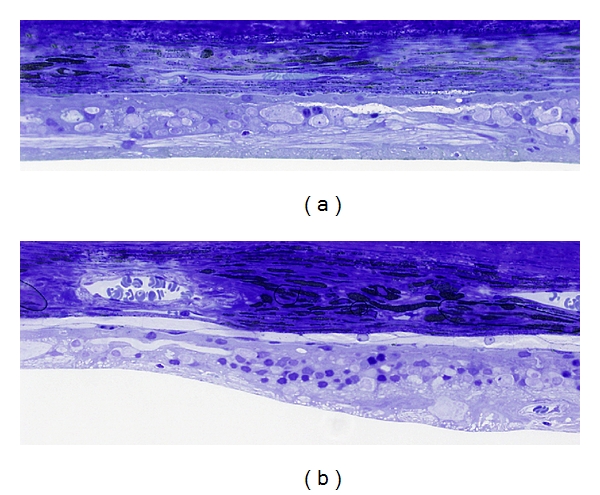
(a) Light microscopy (LM) of the inferior nontapetal retina of the 9-year-old ESS dog. Severe thinning of the entire retina is seen with complete degeneration of photoreceptor cells and inner retinal degeneration, disorganization, and gliosis. (b) LM of superior tapetal retina. A variation in retinal thickness is observed and some areas with a single row of photoreceptor nuclei that are still present. Toluidine blue staining; ×40.

**Figure 5:**
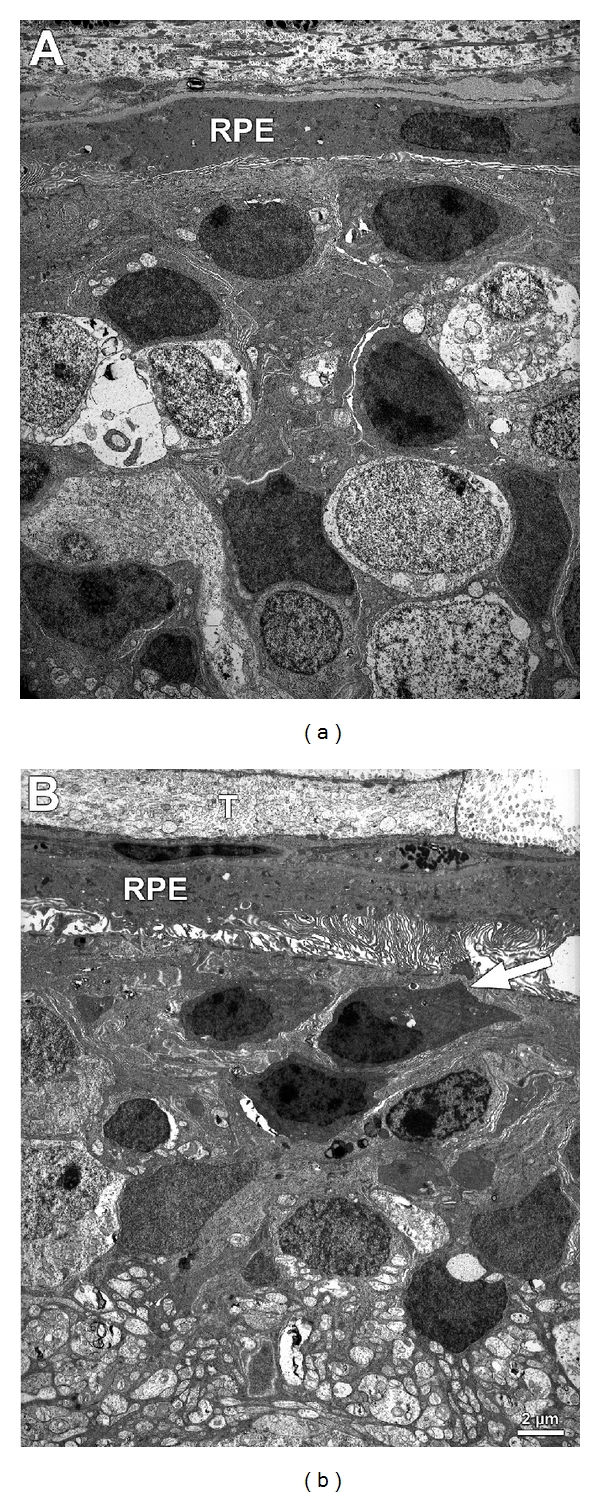
Ultrastructure of nontapetal (a) and tapetal retina (b) from the same dog shown in Figure 4. Note the severely disorganized outer and inner retinal cell layers and structures. The RPE cell layer appears preserved, however. In (b) there is relative sparing of the retina with some minor remnants of photoreceptor nuclei with inner segments (arrow) and an abundance of RPE apical microvilli. RPE: retinal pigment epithelial cells, T: tapetal cells. Bar depicts magnification which is the same for (a) and (b).

In the 9-year-old dog, bilaterally atrophic retinas with no residual photoreceptor cells and sporadic remnants of cells from inner nuclear and ganglion cell layers were observed, with marked retinal gliosis (Figures 4 and 5).

The retinas of the second dog, 6-years-old, showed signs of bilateral generalized retinal degeneration, with complete atrophy of the inferior retina, and a lack of photoreceptor cells in this area, while in the superior retina 1-2 layers of photoreceptor nuclei could be observed.

**Figure 6:**
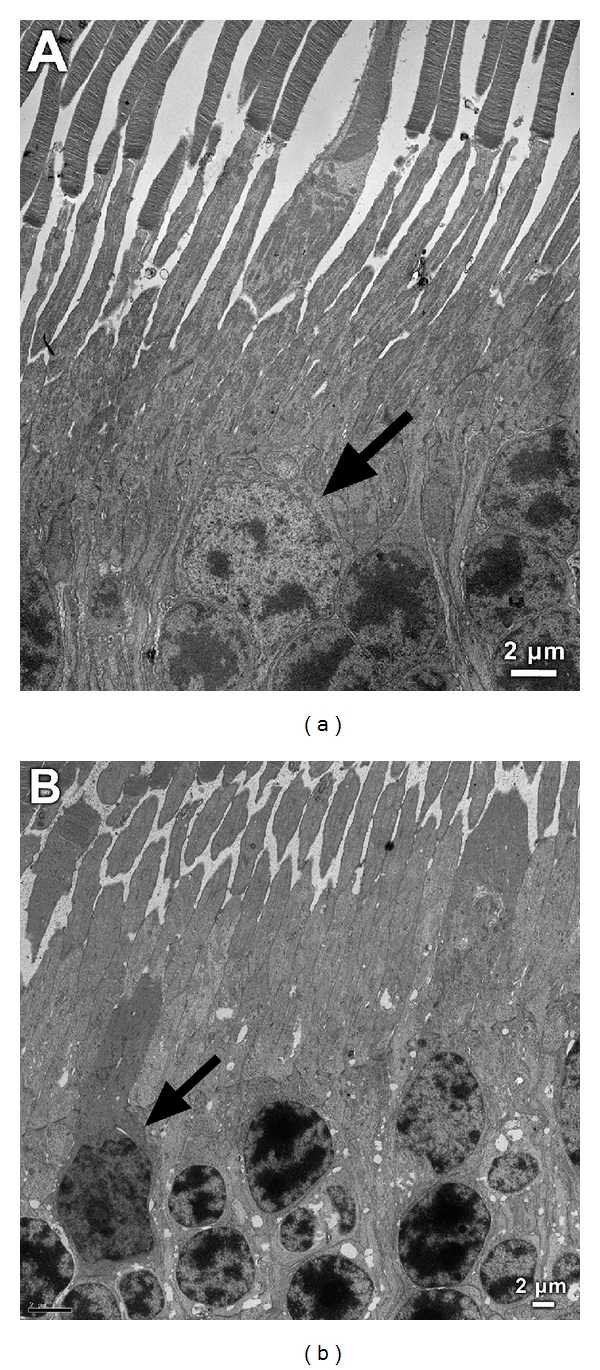
Ultrastructure of the outer retina of a normal 2-year-old dog (a) and that of the affected 3-year-old dog (b). Note the condensed configuration of the cone nuclei in the retina of the affected animal (arrow) in comparison to that of the normal one (in (a), arrow) and the dark appearance of the inner segments in the retina of the affected animal. Bar depicts magnification which is the same for (a) and (b).

The 3-year-old dog, however, demonstrated mainly normal retinal morphology except for changes in the photoreceptor cell layer at all retinal locations examined: cone cell nuclei appeared slightly abnormal, with dense chromatin and with photoreceptor inner segments condensed and shrunken (Figure 6). Photoreceptor outer segments could not be clearly visualized in the thin sections obtained, and thus their ultrastructure was not possible to fully evaluate.

## 4. Discussion

The present study indicates that a majority of the American ESS dogs with hereditary retinal degeneration can be associated with homozygosity for the disease causing allele of the *RPGRIP1* gene. However, four of the 6 dogs homozygous for the *RPGRIP1* mutation had clinical signs of disease while 2 were completely normal appearing both by ophthalmoscopic examination and by ERG. Thus, a clear genotype-phenotype discordance was observed in regards to this group of ESS dogs.

A large proportion of the genetically and/or clinically affected dogs did not show signs of visual impairment until comparably late in life and 2 were clinically completely normal. The reason for the latter finding is unclear. It could be that the *RPGRIP1* insertion by itself is not sufficient to cause retinal degeneration. It appears likely that additional factors are warranted for initiation of photoreceptor cell death such as additional loci involved as modifiers of the disease, as have been described for various forms of PRA, for example, prcd and X-linked PRA [9, 31]. It could possibly also be that there is not full penetrance for the mutation [32].

Similar findings have been observed in comparable human clinical studies in cone rod dystrophies and degenerations. A phenotypic variation between clinical signs in affected individuals and in the onset of hereditary retinal dystrophies has been observed, also by functional testing in human cone rod dystrophies [33]. The variation in clinical signs and time for initial symptoms is especially true for retinopathies caused by the *RPGRIP1* mutation, on a variable genetic background, such as is the case in the human population. In purebred dogs, however, a more uniform phenotype is usually expressed, due to a more homogenous background. This fact is true for most forms of PRA; however, for the cone-rod dystrophies observed in the longhaired and shorthaired Dachshund breeds, with the *RPGRIP1 *and *NPHP4* mutations, respectively, severe heterogeneity has been described for both [22, 27, 34].

In retinal degeneration of the ESS dog clinical signs most often appear comparably late in life and are often difficult to evaluate by the owner. For most of the affected dogs observed in the present study the owners had not noted any visual impairment. The owner of an 11-year-old dog from the present study described that the dog could still play with a transparent frisbee and could easily walk down an indoor stairway in low-lighting conditions. This dog had moderately advanced retinal degeneration with ophthalmoscopically visible changes and was homozygous for the *RPGRIP1* mutation. Another dog showing clinical signs of retinal degeneration had been diagnosed as ophthalmoscopically normal by a veterinary ophthalmologist one year previously, at the age of 7 years. ERGs showed, however, severely reduced retinal function in accordance with a cone rod dystrophy.

It is likely that the cone affection in the disease is early onset, and a defect the dog learns to live with as long as its rod function is normal. Morphology of one 3-year-old ESS dog, homozygous for the *RPGRIP1* mutation, showed ultrastructural changes specifically in cones, while rod photoreceptors were still normal appearing. It is possible that the visual problems become apparent clinically later in life when also the rods start to degenerate. Further, this second phase appears to occur at a variable time point, but most often not until late in life, then leading to a rather fast generalized severe retinal degeneration (retinal atrophy).

ERG recordings proved to be useful in the masked study of the present investigation for objectively detecting reduced photoreceptor function in accordance with cone rod dystrophy due to the *RPGRIP1* mutation. In two cases, however, function in genetically affected individuals was found to be within normal limits. These discordant clinical results could be consistent with incomplete penetrance for the mutation, but other factors previously outlined may also be involved.

Other affected genes or other mutations in *RPGRIP1* may also be present in the ESS breed. One mutated gene, prevalent in at least 32 canine breeds, is prcd, (http://www.optigen.com/, 2011). This could also be a candidate gene since it is known to affect the English and American Cocker Spaniel dog breeds, distant relatives to the ESS dog (Liz Hansen, personal communication 2006). Some of the other mutations causing primary photoreceptor degenerations in dogs are ADAM9 [35], CCDC66 [36], CNGB3 [37], RD3 [38], RHO [39], RPE65 [40], VMD2 [41], PDE6beta [42], PDE6A [43], and PDC [44].

Among the ten PRA cases in ESS dogs previously diagnosed in Sweden, from which DNA was available for the current study, only two individuals were homozygous for the disease causing allele at the *RPGRIP1* locus. Four were found to be heterozygous and four were homozygous for the normal wild-type allele (Table 1). The two cases being homozygous for the RPGRIP1 insertion were diagnosed with PRA at two and four years of age, respectively. Although the number of cases is small there appears to be a tendency for a later onset of PRA among the other eight cases. None of the normal dogs were found to be homozygous for the RPGRIP1 insertion in the Swedish samples, but to make any inferences about the penetrance, a much larger data set would be needed. Taken together, the study of the Swedish samples suggests that at least one more gene is responsible for PRA in the Swedish population.

The complete association between *RPGRIP1* and PRA observed in the ESS thus remains to be fully elucidated. There are strong indications that the *RPGRIP1* gene is involved in the cone rod dystrophy described herein, but the genotype-phenotype discordance observed shows that the genetic background most probably is more complex than previously suspected. In conclusion, there are strong indications that other mutations or modulating genes may be involved in cone rod dystrophy of ESS dogs and could also be causative to other types of hereditary retinal degenerations in the breed. Further investigations in regards to additional loci or genes required for development of cord1 are therefore warranted.

An important goal for vision research is to provide effective treatments for the millions of people affected with retinal blinding disorders. Therapeutic intervention using large animal models such as dogs and cats are effective and necessary methods to utilize before proceeding with human treatment trials. Proof of principle was obtained through therapeutic studies using gene therapy in a dog model of LCA [45], resulting in successful restoration of vision. Similar procedures were performed in human patients with successful outcome [46, 47]. Another promising therapeutic method for retinal blinding disease is stem cell therapy, with or without combination of gene therapy [48]. In preparation for such studies it is, however, of utmost importance that the animal model with its specific retinal disease is precisely characterized clinically beforehand, and with molecular methods, for maximal outcome in the translational process.

## Figures and Tables

**Table 1:** Swedish ESS dogs with genotype-phenotype correlation in regards to the RPGRIP1 mutation. The sex of the dogs is indicated as M for males and F for females. The age of diagnosis is shown as years (y) and months (m) when dogs were first examined and diagnosed with PRA. For the dogs without PRA (normal), the most recent examination dates were used for age at examination. The genotypes are given as −/− for genetically affected, +/− for carriers, and +/+ for genetically clear individuals of the 44 bp deletion in exon 2 of the RPGRIP1 gene.

Dog number	Sex	Age at examination	Phenotype	RPGRIP1 genotype
ESS-008	M	2 y 4 m	PRA	−/−
ESS-014	F	3 y 8 m	PRA	−/−
ESS-004	F	5 y 9 m	PRA	+/−
ESS-006	M	5 y 7 m	PRA	+/−
ESS-016	M	7 y 3 m	PRA	+/−
ESS-020	M	9 y 8 m	PRA	+/−
ESS-003	F	5 y 4 m	PRA	+/+
ESS-005	F	3 y 1 m	PRA	+/+
ESS-007	M	11 y 4 m	PRA	+/+
ESS-009	M	4 y 6 m	PRA	+/+
ESS-015	F	8 y 3 m	Normal	+/+
ESS-017	F	5 y 11 m	Normal	+/+
ESS-018	F	12 y 1 m	Normal	+/+
ESS-019	M	7 y 10 m	Normal	+/+

**Table 2:** Details in regards to dogs in the masked ERG study. The genotypes are given as −/− for genetically affected, +/− for carriers, and +/+ for genetically clear individuals of the 44 bp deletion in exon 2 of the RPGRIP1 gene. The age at the time for ophthalmic and ERG examinations is given as years (y) and months (m).

Dog number	RPGRIP1 genotype	Age at ERG	ERG results	Other clinical findings
1	−/−	9 y 2 m	Abnormal	Normal vision, fundus normal
2	+/−	8 y 7 m	Normal	Normal vision, fundus normal
3	+/−	7 y 9 m	Normal	Normal vision, fundus normal
4	+/−	7 y 9 m	Normal	Normal vision, fundus normal
5	+/−	13 y 10 m	Normal	Normal vision, fundus normal
6	+/−	11 y 11 m	Normal	Normal vision, fundus normal
7	−/−	9 y 2 m	Abnormal	Normal vision, fundus abnormal
8	−/−	9 y 2 m	Abnormal	Normal vision, fundus abnormal
9	−/−	8 y 4 m	Normal	Normal vision, fundus normal
10	+/−	9 y 4 m	Normal	Normal vision, fundus normal
11	+/−	8 y	Normal	Normal vision, fundus normal
12	+/+	9 y 3 m	Normal	Normal vision, fundus normal
13	−/−	12 y 4 m	Abnormal	Reduced vision, fundus abnormal
14	−/−	7 y	Normal	Normal vision, fundus normal

**Table:** (a)

Response	Wave	Normal			Affected		
		Median	5th	95th	Median	5th	95th
S1	b	29.8	16.7	53.1	20.4	15.0	34.0
S2	b	61.6	40.2	101.3	37.7	35.4	45.0
S3	b	98.4	73.9	116.3	52.1	39.4	61.2
S4	b	122.8	78.8	146.4	56.2	50.9	71.2
S5	b	137.3	86.5	185.0	75.0	53.4	85.8
Ssd	a	128.1	102.1	181.9	29.0	16.9	64.9
	b	196.5	138.7	261.6	146.5	72.5	186.0
Sh	a	162.3	137.4	236.8	38.1	23.5	86.9
	b	240.5	171.7	307.7	169.4	77.6	230.8
Psd	a	8.3	3.9	11.5	3.0	2.5	3.6
	b	30.8	13.8	35.0	15.4	6.6	24.9
Pfl	b	34.1	15.4	47.5	15.0	6.9	22.6

**Table:** (b)

Response	Wave	Normal			Affected		
		Median	5th	95th	Median	5th	95th

S1	b	63.5	57.2	87.1	81.0	65.7	87.7
S2	b	75.8	69.2	92.4	84.5	78.5	91.7
S3	b	78.7	75.0	92.3	81.4	76.9	89.8
S4	b	82.3	73.6	95.3	80.3	76.3	96.0
S5	b	80.6	74.8	94.9	86.2	77.3	94.3
Ssd	a	14.3	12.5	15.4	13.5	12.3	15.7
	b	32.7	31.3	59.5	196.5	138.7	261.6
Sh	a	12.6	11.3	13.7	13.3	10.1	15.0
	b	46.9	32.6	52.4	240.5	171.7	307.7
Psd	a	10.6	10.0	12.0	10.0	9.0	12.8
	b	21.6	20.0	23.5	30.8	13.8	35.0
Pfl	b	21.5	20.4	24.8	34.1	15.4	47.5
